# Innate releasing mechanisms and fixed action patterns: basic ethological concepts as drivers for neuroethological studies on acoustic communication in Orthoptera

**DOI:** 10.1007/s00359-018-01311-3

**Published:** 2019-01-07

**Authors:** Bernhard Ronacher

**Affiliations:** Behavioural Physiology Group, Department of Biology, Humboldt-Universität zu Berlin, Philippstraße 13, Haus 18, 10099 Berlin, Germany

**Keywords:** Acoustic communication, Central pattern generators, ‘Genetic coupling’, Coevolution, Sender–receiver evolution

## Abstract

This review addresses the history of neuroethological studies on acoustic communication in insects. One objective is to reveal how basic ethological concepts developed in the 1930s, such as innate releasing mechanisms and fixed action patterns, have influenced the experimental and theoretical approaches to studying acoustic communication systems in Orthopteran insects. The idea of innateness of behaviors has directly fostered the search for central pattern generators that govern the stridulation patterns of crickets, katydids or grasshoppers. A central question pervading 50 years of research is how the essential match between signal features and receiver characteristics has evolved and is maintained during evolution. As in other disciplines, the tight interplay between technological developments and experimental and theoretical advances becomes evident throughout this review. While early neuroethological studies focused primarily on proximate questions such as the implementation of feature detectors or central pattern generators, later the interest shifted more towards ultimate questions. Orthoptera offer the advantage that both proximate and ultimate questions can be tackled in the same system. An important advance was the transition from laboratory studies under well-defined acoustic conditions to field studies that allowed to measure costs and benefits of acoustic signaling as well as constraints on song evolution.

## Introduction

This essay is devoted to the history of investigations on acoustic communication systems of insects, with a main focus on grasshoppers, crickets and katydids, and some borrowing from frogs. I will highlight some major questions, principal conceptual and experimental approaches since the early twentieth century, and their roots in basic ethological concepts. This is also a history of technical developments and how they influenced theoretical ideas. Some controversies about experimental tests of influential concepts will also be discussed.

Grasshoppers, crickets and katydids use species-specific acoustic signals to attract and to identify their mates. Grasshoppers produce sound by moving a file on the hind legs against a vein on the forewings, a process called stridulation, while crickets and katydids stridulate by rubbing their forewings against each other (Bennet-Clark 1998, 1999; Gerhardt and Huber 2002). The basic communication scheme comprises the production of sound by one sex, usually the males, and detection and recognition of the song by the other sex, the females, that approach the singing male phonotactically. Pivotal for a successful communication is the match between signal features and receiver characteristics, and a central question is how this match can be maintained during evolution and species separation (Alexander 1967; Bradbury and Vehrencamp 1998; Gerhardt and Huber 2002).

Before coming to this aspect, in a first chapter I will mention early observations on stridulatory behavior and their connections to influential ethological concepts. Then the neuronal basis for the production of acoustic signals will be covered, in particular the search for central pattern generators that govern the stridulatory movements. The next major question is how the receiver systems may be organized to detect the relevant signals amidst a multitude of irrelevant signals. How are communication signals processed and filtered within the receiver’s central nervous system (CNS)? Careful observations in combination with behavioral experiments were essential preconditions to ask the right questions in neurophysiological studies. An influential idea in this context was that the receiver might compare the incoming signal pattern with a stored ‘template pattern’ in a way like a cross-correlation. In particular, a very early hypothesis, borrowing on the reafference principle (von Holst and Mittelstaedt 1950), assumed that this template could be provided by the central pattern generator for stridulation which might send a kind of ‘efference copy’ or corollary discharge to the pattern recognizing network: “The motor movements necessary to produce stridulation will leave an ‘Efferenzkopie’ in the central nervous system, and the ‘Afferenz’ resulting from stimulation by song will be compared with this, thus enabling discrimination between the songs to be realized” (Haskell 1956, p. 774). This hypothesis guides us to the pivotal question of how a match between sender and receiver properties can be maintained in spite of evolutionary changes. The following sections are devoted to the benefits and costs of acoustic communication and constraints on song evolution—I will highlight a few exemplary field studies, as well as limitations imposed on long distance signaling by signal degradation and noise.

## Innate releasing mechanisms and fixed action patterns as basic ethological concepts

In the 1930s Konrad Lorenz and Nicolaas Tinbergen developed important ideas of classical ethology: the concepts of innate releasing mechanisms (IRMs), sign stimuli, and of fixed action patterns (Instinkthandlungen). In relation to IRMs, Lorenz used the term “Angeborenes Auslösendes Schema” combined with an allusion to a ‘neural filter’ that selects a few relevant stimuli among an abundance of sensory messages, or a stored ‘template’ that is used for a comparison with incoming sensory messages (Lorenz 1935). Later Tinbergen changed the term into “Angeborener Auslöse-Mechanismus” (AAM = IRM = innate releasing mechanism), and with it also to some degree the meaning—see Schleidt (1963). Remarkably, Lorenz and Tinbergen also discussed that ‘releasing’ could be implemented as the removal of an inhibition—as later has been proposed for the release of singing in crickets (Huber 1955; Heinrich et al. 1998). The concept of “instinct” as congenitally preformed patterns of behavior was important to direct attention towards the phylogenetic origins of behavior. IRMs and fixed action patterns were first investigated mainly in birds and fishes (Lorenz 1935; for reviews see: Tinbergen 1952; Schleidt 1963). There was a surge of papers around the fifties and early sixties but later the enthusiasm on IRMs and fixed action patterns in vertebrates tended to fade away, probably due to difficulties to discern innate and learned behavioral components. In contrast to, e.g., birds, acoustic communication of Orthoptera offers the special advantage that it is innate, based on “hard-wired” neuronal structures.

At the onset of the twentieth century, based for example on Friedländer’s experiments on earthworms, rhythmic movements were thought to be governed by a series of chain reflexes (Friedländer 1894; Sherrington 1906). By sophisticated surgical elimination experiments, Erich von Holst demonstrated the existence of a central coordination mechanism (zentrale Koordination) for the rhythmic movements of earthworms and centipedes or the fins of fishes (von Holst 1937, 1939), in anticipation of the central pattern generator (CPG) concept. About 20 years later the term CPG was coined and the existence of CPGs was demonstrated for crayfish swimming and locust flight in deafferented animals (Hughes and Wiersma 1960; Wilson 1961), and later also for the stridulation of crickets and grasshoppers (for a review on CPG history see Mulloney and Smarandache 2010).

The stridulatory behavior of Orthopteran insects is highly stereotyped and species-specific. It is obvious that both signal production and signal recognition are innate capacities, since in temperate zones the offspring of grasshoppers and crickets has no temporal overlap with the parent generation. The sound signals can—nowadays—be easily recorded, analyzed, and modified to perform playback experiments with model songs in which specific parameters are systematically varied, and the response behavior of the receivers to these stimuli can be observed and quantified. Several species can be bred in captivity and it is possible to apply surgical operations, to record muscle potentials and even to record the activity of single neurons during behavioral actions (Elsner 1974, 1975; Hedwig 1992). An additional convenience of insects is the fact that many of their neurons can be individually identified on the basis of their characteristic morphology. These advantages made Orthopteran insects excellent subjects for elucidating the neuronal basis of the ethological concepts of ‘fixed action patterns’ and ‘innate releasing mechanisms’.

### Early observations on acoustic communication in crickets and grasshoppers

Johann Regen in Vienna showed in 1913 in a clever experiment that female crickets were attracted by the sound of a singing male transmitted via a telephone. Thus, this attraction was independent of visual, olfactory or tactile cues (Regen 1913). This experiment already highlighted the main purpose of acoustic communication in Orthopterans: sounds are used to find a mate, and it also demonstrated the basic communication pattern: the males produce sounds to attract females and the females approach the singing male phonotactically.

That the sound producing behavior of Orthopteran insects is species-specific and innate was described as early as 1900 in an enthusiastic account by Ludwig Kneissl, a Bavarian priest: “So aber ist es möglich, selbst die Artbestimmung schon mit dem Ohre vorzunehmen ….” (It is even possible to determine the species identity by ear …; B.R.) (p. 41 in Kneissl 1900); innateness: “ein Probieren, eine Übung, eine Nachahmung, ein Lernen oder dergleichen geht niemals voraus. Schon das allererste Mal tönt uns das fertige spezifische Signal entgegen.” [trial, practice, imitation, learning, and such, never precedes. Already the very first time (after the final molt, B.R.) the final specific signal is produced; B.R.] (p 50); indicating “daß die Lautäußerungen der Heuschrecken auf erblichen Dispositionen und Mechanismen beruhen.” [that the sound production of grasshoppers depends on hereditary dispositions and mechanisms] (p 52). Kneissl further wrote that the songs are used as signals that attract other males and females but that specific odors obviously also contribute to mate recognition in grasshoppers and katydids. Unfortunately, this insightful work, published in German and in a local journal, had not the impact it deserved—similar as the epoch making paper on instinct by Douglas A. Spalding (1873) that was rediscovered only in the 1950ties.

The first descriptions of the communication signals used verbal paraphrases such as “zräzräzräzräzrä”, “drrdrrdrrdrrdsch”, “schirr”, “sräsräsräsrä dschsch” to describe the sounds produced by various grasshoppers (Faber 1929; Jacobs 1953). Tape recorders were a major technical advancement, which allowed for recording, play back and visualization of sounds (e.g., Loher 1957). The first ones, for example, the ‘Tolana’-recorder that Loher used, or the famous UHER recorders, had the disadvantage that they were developed for reporters and musicians, wherefore their upper frequency range ended at 18 or 20 kHz—no problem for cricket recordings but excluding the ultrasound frequencies produced by many grasshopper and katydid species. Later, for example, the very expensive RACAL recorders together with Bruel and Kjäer-microphones and sound level meters solved the problem of ultrasound recordings but at the cost of size and weight. Racal recorders were, in principle, specified as portable but with a net weight of almost 20 kg rather heavy equipment for field studies—a stark contrast to the present days’ equipment with a 1-kg laptop.

## Exploring ‘fixed action patterns’ on the sender side

In Germany, Franz Huber (1925–2017) was a pioneer in neuroethology. He began his studies in 1947 in Munich and started in 1951 his PhD devoted to the search for the cricket’s neural centers for singing, with Werner Jacobs as supervisor. Huber succeeded in eliciting singing in crickets by inserting sharpened needles into certain areas of the brain (more precisely: in the protocerebrum). By changing the location of the lesions he could induce different song types: calling, rivalry, and courtship songs. His dissertation soon became a classic neuroethological paper (Huber 1955). Later, Huber applied electrical stimulation onto the cricket’s brain (Huber 1960), a method refined in grasshoppers and crickets by Berthold Hedwig (1986, 1992). Huber used surgical experiments to infer which parts of the nervous system contribute to produce the song patterns (Huber 1955, 1960). However, for a profound comprehension of stridulatory behavior, it was essential to analyze in detail the fast movements of legs or wings. High speed cameras were loud and therefore not suited for a combined recording of sound and movement. A first approach by Norbert Elsner used the Hall effect to monitor stridulatory movements of grasshoppers. This was an improvement but at the cost of laborious manipulations on the animal: the Hall generator had to be fastened on the thorax, and small magnets on the hind legs—it was by no means certain that animals showed stridulation behavior after such extensive manipulations (Elsner 1970). A breakthrough was the development of the “position detector”. This is an opto-electronic device that focuses light reflected from a Scotchlite sheet attached to, e.g., the grasshopper’s hind leg onto a two-dimensional photodiode (von Helversen and Elsner 1977). This device allowed simultaneous registration of sound and movements at high-resolution, concerning both temporal as well as spatial resolution, with minimal manipulation and restraint of the animals.

**Fig. 1:**
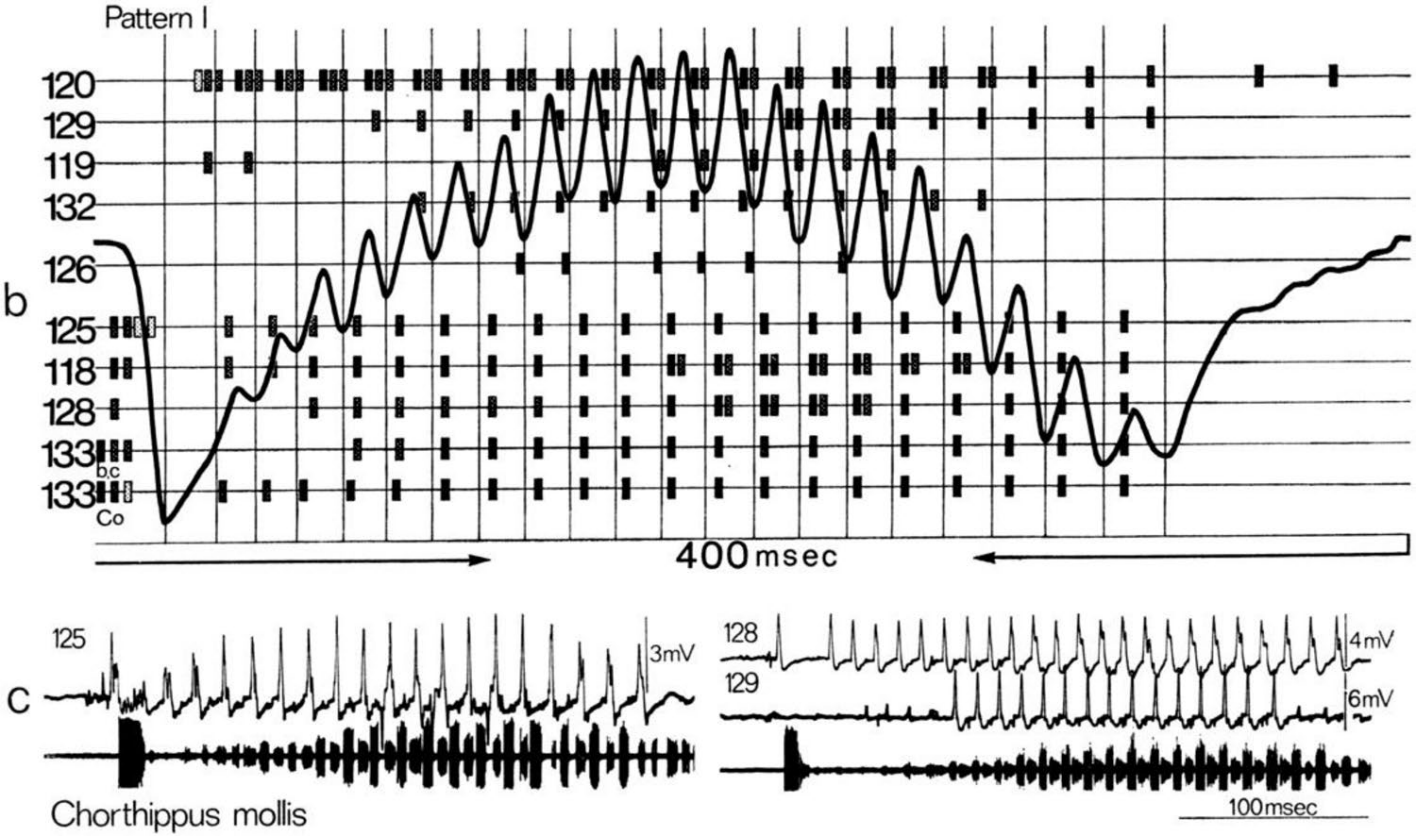
*Chorthippus mollis*: motor activity underlying stridulation. **b** Motor score of a chirp in pattern I; the curve represents the leg position as recorded by the position detector, bars indicate the timing of action potentials in different muscles (120–133). **c** Activities of three muscles during the production of sound pattern I (lower trace); 125 first pleurocoxal muscle (depressor); 128 2nd basalar muscle (depressor); 129 subalar muscle (elevator). From Elsner (1975, p. 313)

Norbert Elsner combined this technique with recordings of muscle action potentials and published famous scores of the timing of up to 20 different muscles that caused the stridulatory movements (Fig. 1; Elsner 1974, 1975; Elsner and Popov 1978). An advantage of muscle recordings in Orthoptera is that the muscles are driven 1:1 by motoneuron action potentials, hence the muscle potentials opened a “window into the CNS-activity” long before recordings from single nerve cells were feasible. In addition, they allowed—indirectly—monitoring the activity of many neurons in parallel, an important goal of Neurobiology.

### The search for the stridulation central pattern generator (CPG)

Where within the central nervous system (CNS) is the stridulation CPG located? As a first step to narrow down the respective structures, Huber applied surgical experiments and postulated a hierarchy in which the brain and the mesothoracic ganglion act together to control the cricket’s stridulatory movements (Huber 1960). Later, this picture was elaborated using various methods (for a detailed account see Elsner 1994). By injecting direct current, Hedwig was able to elicit normal song in the isolated complex of meso- and metathoracic ganglia in the grasshopper *Omocestus viridulus*, thus localizing the CPG for stridulation within these segments (Hedwig 1986). Longitudinal splitting of the meso- and metathoracic ganglia resulted in almost normal stridulation patterns on both sides, but the left–right coordination of the song subunits was impaired (Ronacher 1989). This result, among others, demonstrated that the exact movement pattern is not delivered by the brain’s commands and suggested that in grasshoppers both sides of the metathoracic ganglion house a hemiganglionic CPG for stridulation. The coordination of both CPGs then must depend on commissures within the metathoracic ganglion (Ronacher 1989; for a similar concept for the flight pattern generator see; Ronacher et al. 1988).

Berthold Hedwig succeeded in developing a preparation in which he could record intracellularly from identified neurons while a grasshopper executed stridulation movements, induced by electrical brain stimulation. Together with Siglinde Gramoll he described many stridulatory interneurons located within the metathoracic ganglion complex, many of them confined to one hemiganglion, others connecting the two sides, thus providing nice support for the idea of hemiganglionic pattern generators connected via commissures (Hedwig 1992).

Later, Hedwig went a step upstream in the hierarchy and explored the stridulation command system. He identified ‘command fibers’ descending from the brain whose activity starts the thoracic CPG (Hedwig 1994). As predicted (Otto 1971; Bentley 1977), these neurons exhibit a tonic-spiking activity without any temporal pattern, and their activity is both sufficient and necessary to initiate stridulatory leg movements, thus conforming to the strict criteria for command neurons established by Kupferman and Weiss (1978). These studies, among others, revealed a general hierarchy of motor systems: command neurons drive CPGs that coordinate species-specific movement patterns. However, although the existence of CPGs has been demonstrated by eliminating all afferents, a CPG does not normally operate in isolation, and can be influenced in various degrees by sensory feedback.

Command neurons for stridulation have been identified in the cricket’s brain as well (Hedwig 2000). The stridulation CPG of crickets was long thought to reside in the mesothoracic ganglion which houses the motoneurons for wing movements (Huber 1960; for review:; Kutsch and Huber 1989); then a contribution of the metathoracic ganglion was acknowledged (Hennig 1990). Very recently, application of the “stone-age” technique of cutting connectives and splitting ganglia has quite unexpectedly revealed that important parts of the cricket’s stridulation CPG reside in the abdominal ganglia (Schöneich and Hedwig 2011, 2012; Jacob and Hedwig 2016). A neuron ascending from the third abdominal ganglion to the thoracic ganglia in combination with a neuron descending from the metathoracic ganglion are essential elements of the rhythm generating network as demonstrated by their rhythm resetting capacities (Schöneich and Hedwig 2012).

What might have been evolutionary precursors for the song pattern generator? For grasshoppers it has been proposed, on the basis of similarities in the neuronal patterns, that the stridulation CPG might have its roots in an evolutionary modification of the flight CPG (Elsner and Popov 1978; Elsner 1983). This is an appealing hypothesis but the stridulatory interneurons described so far seem not to be involved in generating the flight rhythm (Hedwig 1992; Elsner and Wasser 1995). Similarly, intracellular studies in crickets gave no evidence for neurons with shared functions in both behaviors (Hennig 1990; see also Schöneich and Hedwig 2012; Jacob and Hedwig 2016). However, the fact that today separate CPGs seem to exist for flight and stridulation does not exclude a homologous origin of both behaviors. Conceivably, a subset of flight generator neurons were originally involved in producing precursors of calls and then, over evolutionary time, were separated in a network dedicated to song production.

**Fig. 2:**
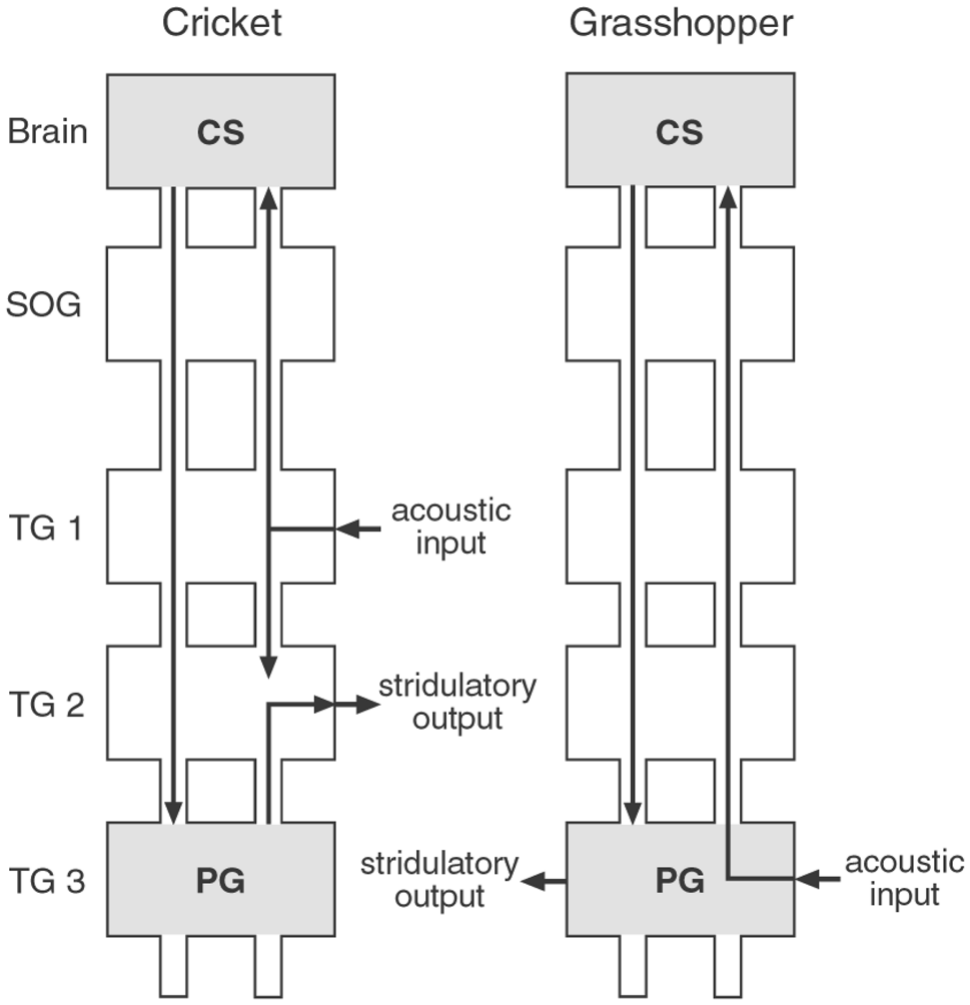
Diagrams of the cephalic and thoracic ganglia of a cricket and a grasshopper. The locations of command systems (CSs) in the brain and pattern generators (PGs) in the metathoracic ganglion complex as well as stridulatory output and acoustic input are indicated. *SOG* suboesophageal ganglion, *TG1 – TG3* thoracic ganglia (= pro- meso- and metathoracic). Figure with original legend from Gerhardt and Huber (2002, p. 50)

At the end of this chapter, I want to bring together the aspects of motor programs and the initiation of a fixed action pattern via sensory stimuli. The scheme of Fig. 2, by Gerhardt and Huber (2002), summarizes the principal pathways by which the fixed action pattern ‘stridulation’ can be elicited by sound signals in crickets and grasshoppers. The grasshopper’s ears are located in the first abdominal segment and acoustic stimuli are preprocessed within the metathoracic ganglion complex. A series of surgical operations demonstrated that the species-specific recognition mechanism must be localized in the grasshopper’s brain (Ronacher et al. 1986). Several neurons ascending from the metathoracic ganglion transmit auditory information to the brain (Römer and Marquart 1984; Ronacher and Stumpner 1988; Stumpner and Ronacher 1991; Stumpner et al. 1991), where the final decision about a response occurs (Ronacher et al. 1986; Bauer and von Helversen 1987). Descending command neurons then activate the thoracic stridulation CPG by means of tonic excitation (Hedwig 1994).

The ears of crickets are located in the forelegs; neurons within the corresponding prothoracic ganglion perform some preprocessing, in particular lateral inhibition that enhances directional cues (Wohlers and Huber 1982; Wiese and Eilts 1985). The cellular basis of lateral inhibition has been investigated by the powerful technique of photo-inactivating single neurons (Selverston et al. 1985). Only two ascending neurons transmit the auditory information to the brain where a network of neurons is devoted to the analysis of the temporal song pattern (see below and Hedwig 2016). Provided the song rhythm is correct, this network activates descending command neurons that in turn activate the stridulation CPG. However, unlike the scheme in Fig. 2, the cricket CPG is not confined to the metathoracic ganglion since its essential elements are located in the abdominal ganglia (see above and Schöneich and Hedwig 2011, 2012; Jacob and Hedwig 2016).

## Exploring the innate releasing mechanism (IRM): how are acoustic signals processed in the receiver central nervous system?

Which features of the communication signals do the animals attend to in their search for a mate? This can be revealed by dummy experiments—as exemplified by Lorenz and Tinbergen—using song models in which signal features are systematically varied and the animals’ responses are quantified.

### Methodological advances

In species such as crickets, where the female approaches a singing male, it is not that easy to quantify the phonotactic response behavior (Gerhardt and Huber 2002). A technical breakthrough was the ‘locomotion compensator’ developed between 1966 and 1968 at the Max-Planck Institut für Verhaltensphysiologie in Seewiesen—in the sixties and seventies *the* European center of behavioral Physiology and Neuroethology—by Ernst Kramer and Peter Heinecke (Kramer 1976; Weber et al. 1981). The ‘Kramer-treadmill’ consisted of a large sphere (50 cm diameter) that could be moved by servomotors. A female cricket was placed on the North-pole of the sphere and its position was monitored by an infrared camera. The animal was not restrained; as soon as the cricket began to move an analog electronic circuitry initiated compensatory movements of the sphere so that the cricket always stayed at the North-pole. The commands to the servomotors allowed a reconstruction of the phonotactic path (Weber et al. 1981). Later, the same principle was transferred into a miniaturized trackball version with a higher temporal resolution: now a tethered animal moves on a small, light Styrofoam ball suspended by an air stream—for details see, e.g., Schildberger and Hörner (1988), Gras and Hörner (1992). By influencing the spiking activity of identified auditory interneurons during intracellular recording Schildberger and Hörner (1988) were able to reverse the walking direction of crickets—a decisive test in which the manipulation of a single neuron’s activity induced a change in behavior.

In some grasshoppers and katydids, a bidirectional communication (“Wechselsingen”, Jacobs 1953) has evolved: as a rule still the male starts stridulation but in these species the female produces a response song instead of performing phonotaxis. The female’s acoustic response then enables the male to approach the female. This shifts costs—the high predation risk of the phonotactic movements—towards the males (see below and Heller 1992). However, bidirectional communication may also reduce the risk for the males from eavesdropping predators as males need to call only a few times to gauge whether responsive females are around. In species with bidirectional communication, one can record the singing responses of females as the basis for an analysis of the IRM (von Helversen 1972, 1979). Dagmar and Otto von Helversen were among the first that used a fully automated, computer controlled apparatus in which the grasshopper females were stimulated with model songs and the computer detected the acoustic response of females via a microphone, allowing high throughput tests on many animals. By the way, the first generations of computers used (Data General Nova 2 and 2C) were huge apparatus, with hardly any memory (only 17 kbytes!), a weight of over 10 kg, and had to be bootstrapped with punched paper tape.

The grasshopper *Chorthippus biguttulus* became a model for acoustic communication studies starting with the pioneering dissertation of Dagmar von Helversen (1972). Already the first dummy experiments revealed that the amplitude modulation pattern of the songs contains the species-specific information relevant for mate attraction, whereas the carrier frequency content is of secondary significance. Song models consisting of broad band noise were well accepted, provided that the durations of noise ‘syllables’ and interspersed pauses were in a narrow range (von Helversen 1972, 1979; von Helversen and von Helversen 1997). There is extensive evidence also from other insect taxa that signal recognition is based primarily on the temporal pattern of songs and much less on the signal’s carrier frequency content (Stumpner and von Helversen 2001; Gerhardt and Huber 2002; Hennig et al. 2004). Hence, the majority of investigations focused on the processing of the temporal patterns of songs.

### Proposed algorithms for the processing of rhythmic sound patterns

#### Processing in the frequency domain?

A conspicuous feature of the songs of many grasshoppers, katydids and crickets is their periodicity and regularity, expressed as distinct peaks in the Fourier spectrum of sound envelopes. Hence, it seemed a parsimonious solution that these regular patterns might be analyzed in the frequency domain, not in the time domain (Michelsen 1985); for example, by a bank of filters for different envelope frequencies as proposed for vertebrates (Joris et al. 2004). This idea was tested by a simple experiment in the grasshopper *C. biguttulus*. If this type of processing algorithm would be realized one expects that two song models whose patterns share the same Fourier amplitude spectrum but differ in their phases should be treated alike by the insects. A critical test was to compare a song with syllables that bear an onset accentuation to its time reversal (now the accentuation is at the end of the syllables). These stimuli differ only in their phase spectrum but have identical amplitude spectra. The result was unequivocal: the grasshopper females responded well to the stimulus with onset accent and much less to the time reversed stimulus, thus providing clear evidence against a purely spectral processing (von Helversen and von Helversen 1983, 1998; see also Schmidt et al. 2008; Ronacher 2016; Krämer and Ronacher 2018). Evidence against a processing in the spectral domain is also reported for crickets (Hennig et al. 2004).

#### Cross-correlation with a stored template

The regularity of Orthopteran songs fostered another idea, i.e., that the recognition mechanism could rely on a stored ‘internal template’ that is compared with the incoming signal pattern in a kind of cross-correlation. This cross-correlation hypothesis has again been tested with *C. biguttulus* in a simple experiment, by inserting subunits with deviating durations in a model song. This manipulation should have disrupted the cross-correlation with a fixed stored template but did in fact not reduce the attractiveness of the song models; hence the cross-correlation idea has been refuted for grasshoppers (von Helversen and von Helversen 1983, 1998). Similarly, *Teleogryllus oceanicus* crickets responded well to shuffled songs and this has been interpreted as evidence against a cross-correlation mechanism (Pollack and Hoy 1979; for a similar result see Hennig and Weber 1997).

However, in both cases, the argument against a cross-correlation mechanism appears not entirely convincing since it assumes that the internal template involves a long part of the song. If the comparison with an internal template is performed over only a short interval, e.g., the duration of a single subunit, this could explain the positive responses to the above mentioned stimuli.

#### Bandpass filters, resonance, autocorrelation

**Fig. 3:**
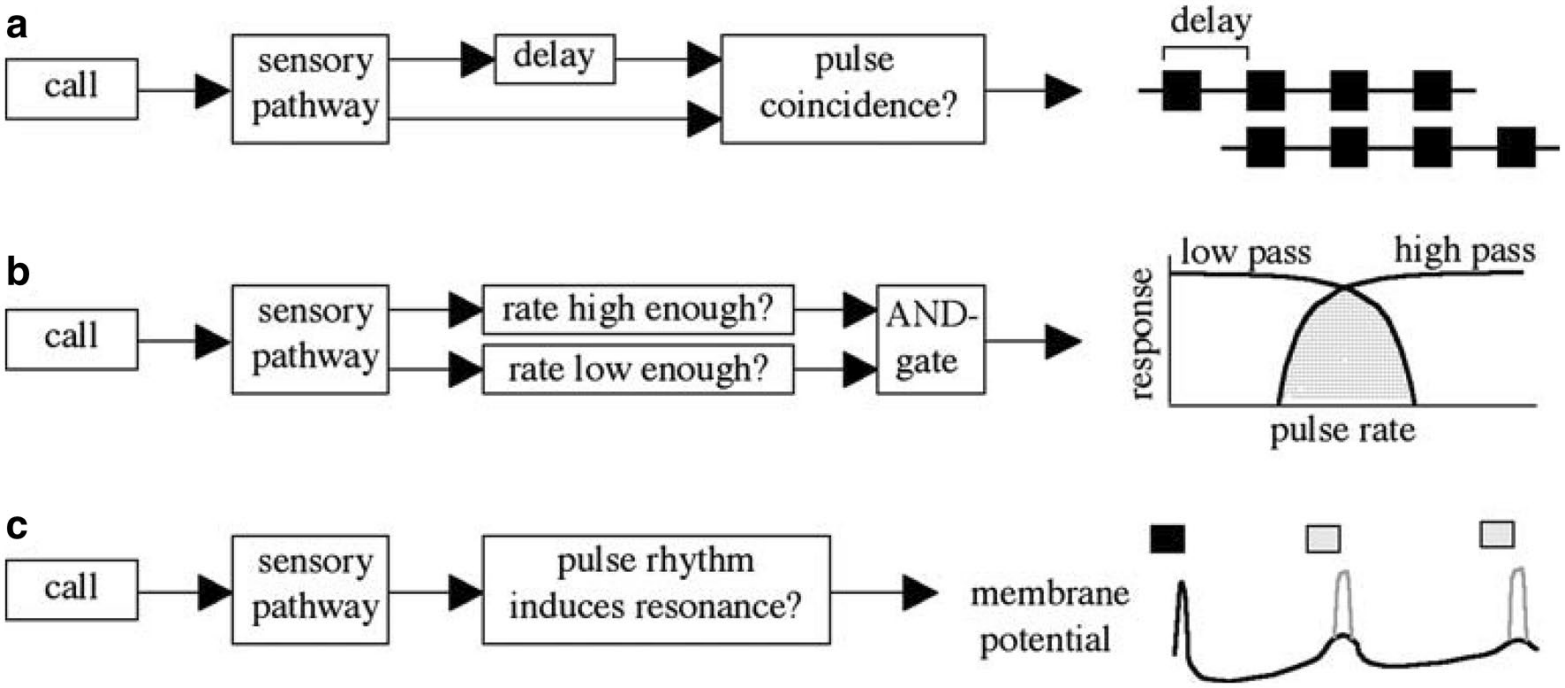
Three models for measuring pulse rate. **a** In an autocorrelation model (left), each pulse travels along one direct and one indirect pathway. The indirect pathway imposes a delay corresponding to the period of the correct rate. The two pathways converge on a coincidence detector. If a call has the correct rate (right), each pulse traveling the indirect pathway will arrive at the coincidence detector simultaneously with the subsequent pulse traveling the direct pathway. **b** In a high/low-pass model, two filters independently evaluate whether the pulse rate is high and low enough (left). An AND gate responds only when both conditions are met. Response functions are shown on the right with the stippled area indicating the pulse rates that pass through both filters. **c** According to a resonance model, neurons respond only when the rhythm of the pulses corresponds to the correct pulse rate (left). Following the initial pulse, oscillations in membrane potential cause increased excitability corresponding to the period of the call (right). If a subsequent pulse (shown in gray) falls during this time of increased excitability, the neuron responds. Any pulses that fall between the rebound excitations fail to stimulate a response. From Bush and Schul (2006, p. 115); with the original figure legend

Cricket songs are comparatively simple, consisting of uniform sound pulses separated by pauses; behavioral tests have shown that many species evaluate primarily the pulse rates or pulse periods (Gerhardt and Huber 2002). Therefore, these systems are well suited to investigate potential processing mechanisms. How can a pulse rate detector be implemented in a CNS? In addition to the idea of a cross-correlation with an internal template introduced above, other models were proposed (Fig. 3). A first model assumes autocorrelation with a delayed path and coincidence detection, another combines low- and high-pass filters, and a third model relies on resonant neurons (Fig. 3, see also Bush and Schul 2006; Schul et al. 2014).

In 1984 Klaus Schildberger, then working in Huber’s lab, described neurons in the brain of *Gryllus bimaculatus* apparently exhibiting low-pass, high-pass and bandpass properties that matched to the behavioral responses (Schildberger 1984). This result soon found its way into textbooks and the bandpass concept became the favored hypothesis of how insects may process temporal patterns.

Interestingly, in the katydid *Tettigonia cantans* behavioral tests with cleverly designed pulse patterns across a wide range of pulse rates gave clear evidence against both the bandpass and the autocorrelation model; instead that data suggested a resonance mechanism (Bush and Schul 2006). This result raised a point of caution against overhasty generalizations of proposed mechanisms across species.

The autocorrelation model (Fig. 3a) postulates that sound pulses are encoded via two neural pathways—a direct one and a delayed, indirect pathway—that converge onto a coincidence detector, an idea first advocated by Reiss (1964). Only if the period of sound pulses matches the delay of the indirect pathway will the coincidence detector be activated and initiate the behavioral response (Weber and Thorson 1989). Such an autocorrelation mechanism has recently been proposed for the cricket *Gryllus bimaculatus* by Berthold Hedwig and coworkers (Schöneich et al. 2015; Hedwig 2016). They described neurons in the brain of the cricket *Gryllus bimaculatus* that exhibit a bandpass response to pulse periods which exactly matches the behavioral responses (Kostarakos and Hedwig 2012). However, this bandpass property is not the result of a combination of low-pass and high-pass neurons but rather the result of a neuronal network in which an excitatory path forms a coincidence detector (neuron LN3) with a rebound provided by neuron LN5 as a delayed excitatory path (Hedwig 2016). Only if the pulse period matches the timing of the postinhibitory rebound, the coincidence detector and the subsequent feature detector neuron (LN4) will spike, and this leads to the observed close match between behavioral response and the neuronal response of LN4. The neuronal network elucidated by Schöneich et al. (2015) thus combines features of the two models shown in Fig. 3 a and c. Strangely, the local brain neurons described by Hedwig et al. are located closely to the axonal arborisations of the ascending neuron AN1 and differ in position and morphology from the neurons reported by Schildberger (1984).

The elucidation of the neuronal network for song pattern recognition in *G. bimaculatus* is a milestone for our understanding of how an IRM may be realized in insects. It has been interpreted as example for an autocorrelation processing mechanism and as evidence against a cross-correlation mechanism relying on a template (Kostarakos and Hedwig 2015; Schöneich et al. 2015; Hedwig 2016). However, it could be argued that the neuron LN5 that provides the inhibitory rebound pattern is in fact an implementation of a ‘template’, admittedly a template on a very short time scale.

### Strength of comparative approaches

In the context of song recognition, the two sibling species *Teleogryllus commodus* and *T. oceanicus* are particularly interesting since they produce relatively complex species-specific songs that exhibit both two different pulse periods. Unexpectedly, female preferences differ clearly between species: for *T. oceanicus* a period filter has been invoked; instead the pulse duration and not the pulse period is crucial for *T. commodus* (Hennig 2003). These species-specific differences of the recognition mechanism were revealed by probing a large region of the song ‘parameter space’; if only stimuli with constant duty cycle are tested, as in most earlier investigations, the different filter types cannot be discriminated (Hennig 2003). The interesting problem is: how can a switch to a different filter type evolve on the basis of likely homologous neural networks in closely related species? An elegant potential solution based on a cross-correlation with an internal template has been proposed by Hennig (2003). In this model, the transition from a period filter to a pulse duration filter is possible by a change in a single parameter, the duration of the evaluation time window; that is, over how many sound pulses the cross-correlation is performed. That a change in a single parameter is sufficient for a transition to a different filter type opened a plausible scenario for the evolutionary separation of the two species.

The American katydid genus *Neoconocephalus* lends itself to trace evolutionary diversifications in song production and song recognition (reviews in Greenfield 1990; Schul et al. 2014). In the ancestral call pattern males produce continuous calls with high pulse rates (150–250 Hz). As derived pattern, in some species the call rate was drastically reduced, whereas other species developed calls with two alternating periods resulting in ‘double-pulses’. A molecular phylogeny of 17 species in this genus revealed that songs with double pulses evolved at least five times independently (Bush and Schul 2010; Schul et al. 2014). Unexpectedly, the corresponding female recognition mechanisms differ substantially between the five species. Females of *N. retusus* and *N. maxillosus* retained the ancestral state and accepted calls with a single pulse rate as well as double pulses or a continuous sound. Females of *N. bivocatus* and *N. triops* paid attention to the double pulse pattern but also accepted a song in which the double pulses were replaced by a single long pulse. In *N. triops*, the call recognition seems to depend on neuronal oscillation, whereas temporal integration is postulated for *N. bivocatus*. In contrast to the previous two species, the amplitude modulation of the double pulses is essential for *N. affinis* females, and double pulses cannot be replaced by a single long pulse (Bush et al. 2009). Schul et al. conclude that in these species “changes in male calls have preceded changes in female recognition, suggesting that males rather than females may lead the divergence of the communication system, and that sender and receiver may evolve more independently of each other than is commonly assumed” (p. 181 in Schul et al. 2014).

### New modeling approaches

A central nervous system has no other information about the external world than the spike trains provided by sensory neurons. However, it is no trivial task to estimate what kind of information a CNS can indeed extract from these spike trains. ‘Reverse engineering’ or stimulus reconstruction methods aim at filling this gap (Rieke et al. 1997). The principle is to stimulate the system with stimuli bearing random amplitude modulations. Part of the resulting spike train data is then used to reconstruct the stimulus in an iterative process from the recorded spike train, by replacing each spike by a filter function; the method’s success is validated on the rest of the data not used to train the algorithm (Machens et al. 2001). This method not only estimates the information transmitted by spike trains, but also reveals what details of an external stimulus are not represented by a specific neuron (Ronacher 2016). An important constraint for the quality of information transmission is the trial-to-trial variability of spike trains caused by the stochastic nature of ion channel opening. This ‘intrinsic neuronal noise’ acts in addition to ‘external noise’ and delimits the discrimination of similar sensory stimuli (Machens et al. 2003; Vogel et al. 2005; Wohlgemuth and Ronacher 2007; Neuhofer et al. 2011). This aspect becomes relevant in the context of sexual selection and female choice where the task is to discriminate between basically similar signals of different conspecific individuals (see Sect. 6).

Most recently, a different modeling approach has been applied to characterize neuronal feature detectors. A ‘LN model’ of feature detectors, based on a *L*inear filter followed by a *N*on-linearity, has been proposed that not only yields an excellent prediction of behavioral responses but in addition also offers a convincing solution for the evolutionary transitions between different recognition filters as found in different cricket and katydid species (Clemens and Hennig 2013; Clemens and Ronacher 2013; Hennig et al. 2014). This model assumes computations on two time scales: a short ‘Gabor’ function with differentiating properties acts as a filter for pulse rate and is combined with a much longer integration time window that monitors the energy of the song. Most remarkably, slight changes in the Gabor filter types will result in behavioral preference functions tuned to either pause duration, pulse duration, pulse period, or duty cycle (Hennig et al. 2014, see also Ronacher et al. 2015). A special appeal of this model is that different Gabor filter types can be easily realized by adjusting the specific timing of excitation and inhibition which may be a way the nervous system implements the corresponding filter functions. These considerations open a convenient bridge to the next section, the match between sender and receiver properties.

## Matching of sender and receiver properties

The exchange of signals for mating purposes depends essentially on a receiver system that is able to detect and to ‘understand’ the signals of the sender. Reciprocally, also the sender is forced to produce signals that can be sensed and interpreted by the receiver. This self-evident statement, however, encompasses two problems:

First, an ideal signal should be not only concise and conspicuous but also uniform, with little variability. But this requirement is hard to realize in poikilotherm insects because of the temperature dependence of neuronal and muscular functions. As a rule, the rhythms of song patterns depend strongly on temperature, which poses a recognition problem for the respective receivers, if their temperature is different from the sender (Walker 1957, 1975; von Helversen 1972; Gerhardt and Huber 2002). Imagine a grasshopper female in a meadow nibbling tender small grasses near the ground, while the male sits in the sun on the top of a grass blade. This situation likely causes a temperature difference between the two of 10 °C or more (Römer 2001). Does the fast song of the hotter male still fit into the lower-temperature female’s ‘expectation’ of species-specific song rhythm, or will this difference abolish the ability to communicate?

The second problem arises when we consider evolutionary changes of the communication signals, for example, at the origin of communication or during speciation events. How can the crucial match between signal traits and receiver characteristics be newly established or maintained in spite of changes? Two hypotheses have been introduced in this context, the ‘genetic coupling hypothesis’ and the ‘coevolution hypothesis’, the pros and cons of which will be discussed below.

### The temperature problem

With respect to the temperature dependence of song patterns, two solutions have been found: (1) to shift the female preference function according to its body temperature, or (2) to evaluate a ratio of two temperature-dependent parameters, and thereby eliminate the temperature influence.

Examples for the first, obviously widespread, solution were found in crickets (Doherty 1985; Pires and Hoy 1992a, b) and in the grasshoppers *Chorthippus parallelus* and *C. montanus* (von Helversen 1979; Bauer and von Helversen 1987). Also, in treefrogs the amplitude modulation rates preferred by the females depend on temperature, and most remarkably, neurons within the torus semicircularis show a corresponding shift in their bandpass properties (Brenowitz et al. 1985; Rose et al. 1985).

Another grasshopper species, *C. biguttulus*, however, has found a solution with extensive temperature compensation. In this species, the female preference function encompasses song patterns produced in a large temperature range since the crucial feature is a ratio between syllable duration and pause duration (von Helversen and von Helversen 1981). Relying on a ratio of two temperature-dependent variables, a female at 35 °C accepts male songs produced between 20° to 40 °C (von Helversen 1979; von Helversen and von Helversen 1994). A simple neuronal implementation for this time-warp resistant recognition capacity has recently been proposed (Creutzig et al. 2009).

Females of two other species of the genus *Chorthippus, C. montanus* and *C. parallelus* show no temperature compensation and shift their song pattern preference according to their body temperature. This enabled a beautiful experiment in search for the location of the pattern recognition network. The head of a female was selectively heated, to about 10 °C above the thorax temperature, and the female’s responses were monitored to songs of males recorded at different temperatures. In this situation, females preferred the male song patterns corresponding to their own head temperature. Since the females themselves showed response stridulation it was possible to compare receiver properties and sender characteristics within one individual. The unequivocal result was that the stridulation pattern was governed by the thorax temperature, whereas the brain temperature was relevant for the recognition process (Bauer and von Helversen 1987).

### Evolutionary matching of sender and receiver properties—genetic coupling or coevolution?

As early as 1956, Haskell has hypothesized that the receiver system may depend on an ‘efference copy’ of the song pattern CPG (See Introduction and Haskell 1956, p. 774, 1958, p. 41: “It is postulated that the discrimination mechanism responsible for distinguishing between various specific songs is closely linked to the inherited motor mechanism which causes typical stridulation in both sexes”). Later, this idea has been expanded and put forth as the ‘genetic coupling hypothesis’: neurons or networks of neurons may be common to both the males’ central pattern generators for song production and to a hypothetical feature detector in females, and these shared elements were assumed to be specified by the same genes (Alexander 1962, 1967; Hoy 1974; Hoy et al. 1977). [Note that the term ‘genetic coupling’ was at that time not used in the sense of genetic linkage]. That hybrid cricket females did prefer the songs of their hybrid “brothers”, suggesting that in hybrids the receiver mechanism is somehow related to the sound production, has been taken as indication for a genetic coupling (Hoy 1974; Hoy et al. 1977). Similarly, a ‘temperature coupling’ of the receiver’s preference function to the temperature-dependent syllable periods of male signals, as observed in crickets, has been taken as additional support for the genetic coupling hypothesis (Doherty 1985; Pires and Hoy 1992a, b). This hypothesis could introduce an attractive mechanism for speciation by hybridization, since the match between sender and receiver will be maintained ‘for free’.

The problem of reciprocal matching between sender and receiver was also investigated in grasshoppers, using hybrids of *C. biguttulus* and *C. mollis* (von Helversen and von Helversen 1975a, b). Since the females in these species do produce response songs (see above), it was possible to investigate both the receiver preferences as well as the produced stridulation pattern in individual hybrid females. Unlike in crickets, the IRM of hybrid grasshopper females was not intermediate and hybrid females tended to prefer the songs of the parental species, not of their hybrid brothers (von Helversen and von Helversen 1975b; see also Finck and Ronacher 2017). Of particular interest for the question of a functional (‘genetic’) coupling were two hybrid females that responded almost exclusively to a *C. biguttulus* song but did themselves produce songs that were *C. mollis*-like. D. and O. von Helversen interpreted these examples as particularly strong arguments against the concept that common neurons were shared between the sender CPG and the receiver mechanism and proposed the ‘*coevolution hypothesis’* as an alternative scenario, that is, a stepwise adaptation between sender and receiver properties due to mutual selective forces (von Helversen and von Helversen 1975b, 1994; for a profound discussion see also Doherty and Gerhardt 1984).

As an additional argument against a functional coupling the spatial separation of the (thoracic) stridulation CPG and the IRM receiver mechanism (located in the head) was emphasized (Bauer and von Helversen 1987; see also Ronacher et al. 1986; Hedwig 1986; Ronacher 1989). In hindsight, however, one must admit that the spatial separation of CPG and IRM alone is not a stringent argument, since conceivably an ‘efference copy’ or corollary discharge—as originally proposed by Haskell—might be provided by neurons connecting the thoracic CPG to the brain. Indeed, in the cricket *G. bimaculatus*, a corollary discharge interneuron (CDI) has recently been described whose ramifications span from the terminal ganglion up to the brain (Poulet and Hedwig 2006a, b).

Although ‘genetic coupling’ and ‘coevolution’ are not mutually exclusive hypotheses, there was quite some skirmish between the major representatives of the two hypotheses. To summarize, the dispute between adherents of the genetic coupling or the coevolution hypothesis had no odds-on favorite, probably until the years 2015/16 when Hedwig and coworkers unraveled the neural network responsible for the temporal pattern recognition in crickets (see above and Hedwig 2016). Hereby it has been demonstrated that the neuronal network for the IRM does not share any common elements with the thoracic/abdominal stridulation CPG, as assumed by the original ‘genetic coupling hypothesis’. Still, we do not know what genes are involved in shaping the specific properties of these neurons, such as endowment with ion channels, synaptic transmitters, connectivities et cetera. “Even if separate CPGs control song and preference, some or all genes may have pleiotropic effects on both” (p 2244 in Wiley and Shaw 2010). These authors recently reported genetic linkages in Hawaiian *Laupala* crickets, a genus that shows an extremely rapid species radiation. In hybrid backcrosses, the genetic linkage produced a strong association between male song pulse rate and the female preferences for pulse rate. The tight coupling between song and preferences implies that evolution acting on one trait would directly induce the corresponding changes in the other, and thus favoring rapid diversification and speciation (Shaw and Lesnick 2009; Wiley and Shaw 2010). On the other hand, the examples of a non-parallel evolution of male calls and female recognition mechanisms in *Neoconocephalus* katydids mentioned above argue against genetic coupling (Schul et al. 2014).

I want to conclude this chapter with a side note: a convincing argument for the capacity of coevolution to induce an astounding concordance between sender and receiver features is the mimicry of orchid flowers. Several orchid flowers exhibit a delicate imitation of the shape and odor of wasp females and thereby seduce male wasps to perform ‘pseudo-copulations’ by which the wasps transfer the pollen from one flower to the next (Paulus and Gack 1990; Schiestl et al. 1999). Evidently, this beautiful match in a sender–receiver relation between plant and insect species is achieved without a basis of common genes. Another striking coevolutionary convergence exists between the tuning of the ears of parasitoid flies and the sound signals of their katydid or cricket hosts (Lakes-Harlan and Heller 1992; Robert et al. 1992).

Lets come back to the outset of this account, and to the legacy of Lorenz and Tinbergen. So far, most examples in this account were devoted to proximate questions, i.e., how certain behavioral performances are implemented in an animal. In the next chapter, I will come back to the starting point and set the focus on ultimate questions—which were one of Tinbergen’s special strengths. He did not only ask “what is a behavior good for”, but also designed experimental approaches to this type of question, which cannot be answered in the lab (e.g., Tinbergen et al. 1962). While most sections presented so far described lab work and theoretical considerations, now it is time to spend some lines on field work.

## Benefits and costs of acoustic communication

Producing sounds is costly; it requires energy and likely involves further costs such as the risk of attracting predators or parasitoids (see below). A first obvious question hence is: do these expenditures pay? This question was investigated in a seminal field study by Helmut Kriegbaum (1988). He compared the mating success in a population of silent *C. biguttulus* males (whose forewings were removed) with the success of a separate population with intact, singing males. Compared to the intact population the mating in the population with silent males was delayed by 1.7 days, but by day 14 post adult molt all females were inseminated, in spite of the absent acoustic stimulation. However, in a mixed population with equal numbers of mute and singing males, the intact males got 95% of the observed copulations notwithstanding the fact that mute males encountered females by chance with the same probability as intact males (Kriegbaum and von Helversen 1992). This result underpins the relevance of acoustic signals for the males’ mating success, even though females may use also other modalities such as visual or chemical cues for the decision of whether or not to accept a potential mate (von Helversen 1986; Kriegbaum and von Helversen 1992; Elsner and Wasser 1995; Finck et al. 2016a, b).

### Energetic costs

A major advantage of acoustic communication is its use for long distance communication in cluttered habitats where vision fails. Many cricket and katydid species produce loud signals that allow communication over tens, in some species over hundreds of meters (Römer 1987; van Staaden and Römer 1998). Long distance communication with sound is, however, constrained by the frequency dependent attenuation of sound. High frequency sounds are strongly attenuated; to transmit sound signals to a distant receiver it would therefore pay to use low frequency sounds (Römer and Lewald 1992). However, for small animals such as insects it is difficult and costly to produce low frequencies (Bennet-Clark 1971, 1998, 1999). With a few exceptions (Bennet-Clark 1999), sound production efficiency—measured as acoustic power/net metabolic power—is low in insects, mostly < 1% (e.g., Prestwich 1994; Prestwich and O’Sullivan 2005). Nevertheless, in the field some phaneropterid katydids do produce up to 100,000 syllables per day or up to 10,000 syllables per hour, indicating that energy expenditure during stridulation may not be a limiting factor in these insects (Heller and von Helversen 1993).

### Costs through predators and parasitoids

Communication in the context of mate attraction involves other costs apart from energy expenditure for signal production: the signals may attract not only mates but also predators or parasitoids (and also rivals or satellite males). On the other side, also a receiver that moves towards the sender experiences costs. The energy expenditure during a phonotactic approach is probably less relevant compared to the predation costs due to the increased conspicuousness of moving. Evidently, it is no easy task to estimate and to compare the respective weights of these costs. While the energy consumption during stridulation can be measured in the laboratory (Prestwich and O’Sullivan 2005) other costs must be estimated in field studies. Here I can mention only a few pioneering studies.

Several katydid species have developed a ‘bidirectional’ acoustic communication, in which the male approaches a female responding to its call (Heller and von Helversen 1986; Robinson et al. 1986). Males of species with responding females show a trend to sing less frequently than males of species where the females approach the male (Heller and von Helversen 1993). Since the two communication types occur in closely related species with similar ecology, it was possible to measure the respective risks associated with calling and phonotaxis in terms of survival rates. In *Poecilimon veluchianus*, a katydid with calling males and phonotactically approaching females, the survival rates of both sexes were similar. In contrast, in the species *P. affinis*, whose males call *and* perform phonotaxis to a responding female, the males’ mortality was higher than that of females, thus demonstrating the high predation risk of a phonotactic approach (Heller 1992). This risk is further underlined by a study of the sex distribution of katydids captured by a Scops owl. Females of *Platycleis albopunctata*, which move phonotactically towards singing males, were captured by the owl four times as often as males—the peak frequency of the male song is beyond the hearing range of this owl (Heller and Arlettaz 1994). Moving mute katydid females are also at higher risk of bat predation than acoustically signaling males (Raghuram et al. 2015).

However, the signal production as well is fraught with risk, since not only females may be attracted by a singing male, but also predators such as passively listening bats or parasitoids (Belwood and Morris 1987). Depending on the bat predation risk, a Panamanian katydid switches between stridulation and tremulation signals (Römer et al. 2010). American, European and Australian katydids and crickets suffer from parasitic flies (e.g., *Ormia ochracea, O. lineifrons, Thereobia leonidae, Homotrixa alleni*) that localize singing males phonotactically (Cade 1975; Robert et al. 1992; Lakes-Harlan and Heller 1992; Lakes-Harlan et al. 1999; Allen et al. 1999; review in; Hedwig and Robert 2014). In an extreme case, the pressure by parasitic flies was so strong that over a few years in Hawaiian cricket the majority of males gave up singing and even reduced their stridulation apparatus within less than 20 generations (Zuk et al. 2006). Remarkably, mute male morphs (‘flat wing males’) continue to silently generate the singing movements (Schneider et al. 2018). However, as mute males find females by approaching calling males, mute male morphs are selected against if too few calling males are present.

## Signal degradation and other constraints for sender–receiver evolution

Many cricket and katydid males produce loud signals to attract females over distances of tens of meters (Römer 1987). Such communication distances do entail different problems for a receiver: (1) to perform phonotaxis, the female must identify the male’s signal in spite of usually degraded signal quality. Not only the sound amplitude is reduced with distance but also the sound pattern will be degraded, e.g., through reflections and echoes (Michelsen and Larsen 1983; Römer and Lewald 1992; Römer 2001). In addition, different kinds of noise, and in particular in dense populations interfering signals from conspecifics and other species may pose problems for signal recognition (Greenfield 1994, 2015; Hartbauer et al. 2005; Römer 2013). (2) Localizing the sound source entails additional difficulties; however, this aspect will not be covered in this review.

**Fig. 4:**
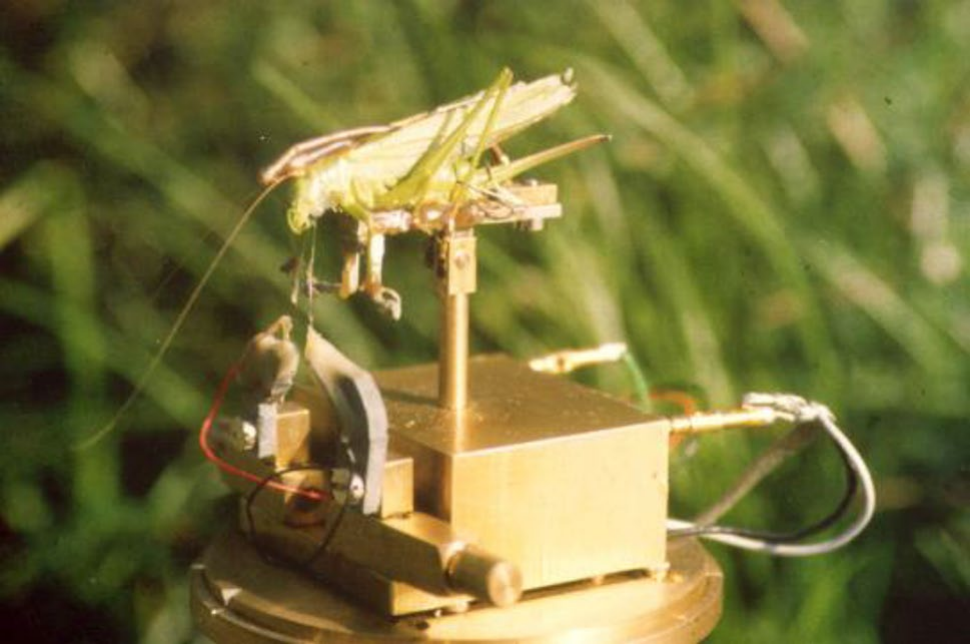
The principle of the “biological microphone”: one or two electrodes are attached to the nervous system to record extracellular action potential activity from identified nerve cells responding to sound. The preparation can be placed in any habitat, and the afferent nervous activity be recorded. For details of the method, see Rheinlaender and Römer (1986), Römer and Lewald (1992). Photo by Heiner Römer

Studies on sensory receptors and interneurons performed in laboratories were not apt to address these problems until Jürgen Rheinlaender and Heiner Römer translated the brilliant idea of a ‘biological microphone’ into a portable device that could be used in the field (Fig. 4; Rheinlaender and Römer 1986). The principle is to record extracellularly the spike activity of identified neurons, e.g., from the prominent local Omega neuron, and thus use the insect’s ears themselves as a microphone. This ingenious method allowed to investigate the communication problems in the field, and to analyze the challenges of sound transmission in natural habitats from an insect’s perspective (Römer and Bailey 1986; Römer and Lewald 1992; for reviews see; Römer 2001, 2013). This method also made it possible to address the basic question of how the conditions of sound transmission in different habitats may constrain trajectories for the evolution of communication signals and receiver systems. Notably, signal degradation induces additional variability of signals arriving at the receiver and therefore aggravates the problem of detecting differences between signals of potential mates and discriminating these from irrelevant information (Wiley 2006, 2013; Ronacher 2014, 2016).

In this context, it needs to be emphasized that not only external conditions such as signal degradation and noise, or the presence of eavesdropping predators and parasitoids (see above) constrain the evolution of signaling but also that senders and receivers exert mutual selective forces on one another. This has been emphasized in an influential paper on ‘sensory drive’ by John A. Endler (1992). Selection will favor signals and behavior that yield a high signal-to-noise ratio and minimize signal degradation. The effectiveness of signals depends not only on signal design and environmental conditions but particularly on the receiver’s sensory equipment. Characteristic features of sensory systems therefore will influence mate choice criteria and these exert a bias for the evolution of male trait properties. Thus, the evolution of sensory systems, signals and communication behavior is tightly coupled and characterized by mutual interdependent influences (Endler 1992). From a receiver’s position, the crucial problem remains to evaluate basically similar signals, in particular, to detect differences between the signals of high or low quality potential mates (Kriegbaum 1989). Noise and signal degradation, as well as the trial-to-trial variability of spike trains may become limiting factors for this type of task (Ronacher and Stange 2013; Ronacher 2014, 2016). Hence, it would be worth to spend some effort not only on the intra- and inter-individual variation of song patterns but also on the interindividual variation of females’ preference functions for calling songs. Finally, we should not lose sight of the fact that a female’s decision does not depend entirely on the evaluation of long distance signals but may include other traits during short distance courtship interactions, such as visual displays (e.g., Elsner and Wasser 1995; von Helversen 1986; von Helversen and von Helversen 1994) or chemical cues (Neems and Butlin 1995; Finck et al. 2016a, b; Finck and Ronacher 2017).

## Outlook on evolutionary trajectories in orthopteran communication signals

The taxa crickets and grasshoppers separated in the Permian, more than 250 million years ago (Song et al. 2015; Greenfield 2016). Cricket songs appear rather simple, consisting of uniform sound pulses, whereas the songs of grasshoppers exhibit much more diverse amplitude modulations patterns. The simple form of cricket sound pulses is likely due to the simple wing closing movement and the resonance mechanism used that allows for high sound pressure levels in a narrow frequency range but at the cost of a slow build up and decay that precludes brief transients (Bennet-Clark 1998). Grasshopper songs are typically less loud and exhibit complex amplitude modulation patterns with transients on short time scales due to the complex leg movements underlying sound production (e.g., Elsner 1974; Elsner and Popov 1978; von Helversen 1986). Katydid songs range between the two taxa in both song complexity and intensity (Heller 1988). Remarkably, there is evidence that 165 million years ago Jurassic katydids already used a resonant stridulation apparatus tuned at 6.4 kHz (Gu et al. 2012).

Did differences between the auditory pathways of various Orthopteran taxa shape the evolution of communication signals? There exists an interesting difference between crickets and grasshoppers. In crickets, only two interneurons dedicated to carry forward auditory information have been found that ascend from the thoracic ganglia to the brain (Wohlers and Huber 1982; Schildberger and Hörner 1988), whereas in grasshoppers up to 20 ascending auditory neurons exist (Römer and Marquart 1984; Stumpner and Ronacher 1991; Stumpner et al. 1991; Ronacher 2014) and a neuronal population code is established (Clemens et al. 2011). The low number of ascending auditory neurons in crickets may be affordable since their cercal system takes over large areas of predator detection; only the avoidance reaction to ultrasound emitting bats is triggered by the ascending neuron AN2 (Wohlers and Huber 1982; Marsat and Pollack 2006). In katydids, there exist at least 4 to 5 ascending auditory neurons (Stumpner and Molina 2006; Triblehorn and Schul 2009; Stumpner and Nowotny 2014). Conceivably, the low number of ascending neurons in crickets may also have constrained the processing of more complex song patterns, whereas for the evolution of sophisticated grasshopper songs the large number of auditory neurons that project to the brain may have been pivotal. Unexpectedly, the morphology and physiology of the ascending auditory neurons in grasshoppers are highly conserved between locusts and gomphocerine grasshoppers, two taxa that were separated for more than 50 million years and differ strongly in the respective significance of acoustic communication (Neuhofer et al. 2008). We do not know what the stabilizing selective forces may have been that prevented an ongoing adaptation to the needs of acoustic communication in grasshoppers—an educated guess is the system’s task of predator detection. In contrast to the grasshopper example, responses of homologous auditory neurons differ between closely related katydid species (genus *Neoconocephalus*), and these differences contribute in establishing species-specific recognition mechanisms (Triblehorn and Schul 2009). Remarkably, a comparison of female preferences with male call traits in *Neoconocephalus* species suggests that males rather than females may have led the evolutionary divergence of these communication systems (Bush and Schul 2010; Schul et al. 2014). These examples underline the powerfulness of a broad comparative approach and the wealth of insights we can find in ‘non-model’ species.

## Abbreviations

CNS: Central nervous system
CPG: Central pattern generator
IRM: Innate releasing mechanism
